# Evaluating the role of dental education in shaping aesthetic preferences and clinical choices in Cambodia

**DOI:** 10.3389/froh.2025.1624308

**Published:** 2025-09-15

**Authors:** Chhean Pisethsathya, Chhim Chamroeun, Phav Samnang, Anand Marya, Abedelmalek Kalefh Tabnjh, Siddharthan Selvaraj

**Affiliations:** 1 Faculty of Dentistry, University of Puthisastra, Phnom Penh, Cambodia; 2 Department of Cariology, Odontology School, Sahlgrenska Academy, Gothenburg University, Gothenburg, Sweden; 3 Department of Applied Dental Sciences, Faculty of Applied Medical Sciences, Jordan University of Science and Technology, Irbid, Jordan; 4 Dental Research Unit, Center for Global Health Research, Saveetha Medical College and Hospital, Saveetha Institute of Medical and Technical Sciences, Chennai, India; 5 Department of Dental Research Cell, Dr. D. Y. Patil Dental College & Hospital, Dr. D. Y. Patil Vidyapeeth (Deemed to be University), Pune, India

**Keywords:** dental students, non-dental students, dental esthetics, patient perceptions, smile

## Abstract

**Aim:**

The aim of this study is to evaluate and compare the factors influencing perceived dental appearance among dental and non-dental university students.

**Methods:**

A questionnaire was provided to all the consenting participants in the study. The survey was validated with a pilot study and the results of the pilot study were not included in the final results. A total of 420 participants were recruited for the study, of which 210 were dental students and 210 were non-dental students. The recruited participants answered questions regarding smile and esthetics perceptions using a validated questionnaire.

**Results:**

Significant differences were observed between groups in perceptions of facial and dental aesthetics, with non-dental students placing more importance on features like nose shape (*P* = 0.038) and white or specific tooth shapes (*P* < 0.05). Dental students showed greater awareness of dental alignment but reported lower overall satisfaction. Non-dental students had higher aesthetic treatment experience and intentions (*P* < 0.05), brushed more frequently, but flossed and visited the dentist less often. Overall, non-dental students had significantly higher satisfaction scores than dental students (*P* < 0.001).

**Conclusion:**

It was observed that both dental and non-dental students had a good awareness on esthetics; however, dental students showed greater sensitivity towards aesthetics due to their deeper knowledge and educational background. On the other hand, non-dental students due to their superficial knowledge regarding dentistry had higher expectations from dental treatment and expressed unrealistic esthetic demands.

## Introduction

Over the last few decades, the demand for improving dental esthetics from patients has greatly increased. In response, dental professionals have developed a variety of techniques to meet patient expectations. Direct and indirect esthetic restorations, tooth whitening, and orthodontic treatment are some of the common procedures available. A patient's natural esthetic has become an essential criterion to consider during treatment (1). Dental beauty has been defined differently across populations, regions, countries, and continents (2). Cultural, ethnic, and racial concepts of beauty may also influence the perception of beauty (3). Therefore, every patient must be treated as an individual (2).

Dissatisfaction with dental appearance is often caused by untreated dental caries, non-aesthetic or discolored anterior teeth, and malaligned teeth. Furthermore, patient self-esteem and quality of life have been found to be increased by improving dental aesthetics (4). When a person's smile is compromised by dental disease or malocclusion, this often leads to a loss of self-esteem and damage to his or her overall physical and mental health (5, 6). According to Langlois et al, in the year 2000 found that, esthetic concerns may be influenced by social media in young adults (7). Physical attractiveness receives more positive judgments and may impact academic performance in both children and adults (7, 8). Studies have shown that dissatisfaction with dental esthetics can have a negative impact on factors such as social competence, quality of life, psychological adjustment, and relationship status (3, 6).

Patients interested in improving their dental appearance frequently demand treatments such as tooth whitening, anterior teeth restorations, labial veneers, crowns, and orthodontic treatment (1, 6, 9, 10). Tooth color, shape, position, restoration quality, and the dentition's general arrangement, especially of the anterior teeth, have been shown to be significant factors affecting the overall dental appearance (4). Previous reports have shown that females are more sensitive than males regarding the appearance of teeth; however older and more highly educated people place less importance on dental esthetics (1, 2, 11). While there is the lack of agreement about which factors influence patients' decisions, there are still many lesser explored factors that influence a patients' decisions regarding the selection of specific types of therapy to improve their dental aesthetics (12). It has been suggested that many factors, such as gender, socioeconomic background, and age, affect self-perception of dental appearance, which means that individuals may interpret the idea of beauty differently (13–15). Dindaroğlu and colleagues in the year 2016, found that the general education level might influence the perception of smile esthetics (12).

Most of the previous studies have been conducted focusing on a specific aspect of esthetics but have not considered the educational background of individuals. Most of the other studies have not considered the other social and demographic factors such as educational background, occupation etc. In Cambodia, no studies have been conducted that consider the cultural ethnicity of the population and the educational background. Many studies did not validate their questionnaires for use in their local populations. Previously conducted studies have tried to compare the esthetic perceptions between dentists and laypersons but without comparison photos, making it extremely difficult to carry out a questionnaire-based survey without visual aids. This is also the first study in Cambodia to not only analyze the perception of educated laypersons but also dentally educated individuals to analyze whether there is any difference in their perception or not (1, 2, 12).

In Cambodia, traditional values, as well as access to dental care and general knowledge about dental aesthetics have also been shaped by social economics and education around healthy behaviors. It is an interesting context to observe how these factors shape dental aesthetic perceptions. Education level is a strong measure of an individual's exposure and understanding of dental health and beauty standards. Higher levels of education generally promote a more global and/or medicalized awareness of beauty standards. On the other hand, lower levels of education may result in individuals relying upon more traditional beauty standards of their culture (2).

This study evaluates the factors that influence Cambodian students' perceptions of dental aesthetics and how these influence treatment decisions and also provide dentists an insight into how individuals with different backgrounds perceive esthetics and how can dentists better treat patients with different esthetic demands. Furthermore, our study also explores the possible correlation of education level and the aesthetic importance and perspective among different population.

## Materials & methods

### Study design

This was a cross-sectional study in which the information was collected using a structured, interviewer-guided questionnaire. The participants were provided information on what kind of data would be collected and after receiving their consent they were requested to collect in a classroom setting for answering the questionnaire.

### Sample size & sample design

The systematic random sampling method was used for selecting participants. We recruited 420 participants of which 210 were University of Puthisastra non-dental students who attended the University of Puthisastra Dental Clinic (UPDC). The other 210 participants were University of Puthisastra dental students from years two to six. The sample size was calculated based on the previous studies (4, 16, 17).

Participants for this study were, UP undergraduate students aged 18 or older who agreed to participate in the study were included in this study. Participants who refused to participate in this study were excluded from the study.

### Data collection

The questionnaire was adapted from previous studies (4, 16, 17). Few questions were altered by the researchers to satisfy the research objectives and for the Cambodia context. The questionnaire was translated from English into Khmer and back-translated to ensure accuracy.

A pilot study was conducted on 15 patients. Problems in understanding and answering the questions were identified, and modifications to the questionnaire made accordingly.

The data was collected using validated questionnaire which were further subdivided into various sub-sections according to treatment needs and esthetic demands. The questionnaire had a 4-point Likert scale with 4 categories for each question: very dissatisfied, dissatisfied, satisfied and very satisfied. The choices ranged from the extreme negative to extreme positive in terms of their experiences and demands and patients were requested to select one of these.

#### Criteria for satisfaction

To measure satisfaction level with dental aesthetics, a 4-point Likert scale was used.

Very Dissatisfied with dental aesthetics (Score = 1): The participants described their dental aesthetics as so unattractive that their dental aesthetics could be perceived to be negatively affecting their confidence or willingness to smile or socialize.

Dissatisfied with dental aesthetics (Score = 2): Participants found their dental aesthetics unattractive because of two or three components, and changing their self-image or comfort with smiling moderately.

Satisfied with dental aesthetics (Score = 3): Participants rated dental aesthetics as generally satisfactory, the participant had some concerns, however, which would not influence daily functioning or social confidence.

Very Satisfied with dental aesthetics (Score = 4): Participant's rated dental aesthetics fair to satisfied, the appearance of the teeth and smile were wholly satisfactory with the appearance desired, there was no variability in seizure or desire for improvements or healthier dental aesthetics.

The privacy and confidentiality of participants was kept, and informed consent obtained. Before conducting the survey, all study participants were informed that the results would be anonymous and that no identifying information would be collected from them at any given point in the survey.

### Ethics approval

Ethics approvals for our study were received from the University of Puthisastra Research Committee (Protocol No:005UPRC).

### Data analysis

The data obtained from this study was entered and analyzed using SPSS version 26, and the significant level was set to 0.05 (5%). The researchers initially performed preliminary data analysis to investigate missing values and wrong data entry as part of the data cleaning process. The statistical analyses employed in the study were descriptive analysis, pearson's chi-square test was performed to determine the association between the participants' perceived importance of an attractive face, perceived beauty, perceived importance of dental aesthetics, perceived importance of appearance, perceived satisfaction with teeth, previous treatment of anterior teeth, oral habits, and treatment intentions with study groups (dental students and non-dental students). If more than 20% of the expected counts are less than 5, we use the Fisher exact test instead of the Chi-square test. We performed an independent t-test to determine the mean difference in satisfaction scores between the study groups and gender. An ANOVA was performed to determine the mean difference in satisfaction scores between economic statuses. In addition, we conducted Pearson's correlation analysis to examine the relationship between age and satisfaction score.

## Results

A total of 420 respondents participated in the study (Table 1), with equal proportions of dental students (*n* = 210) and non-dental students (*n* = 210). For the dental students, they had a mean age of 22.07 (SD = 2.66), and the majority of them were males (*n* = 128, 61.0%) and earn below 350 USD per month (*n* = 188, 89.5%). For the non-dental students, they had a mean age of 21.16 (SD = 1.20), and the majority of them were females (*n* = 134, 63.8%) and earn below 350 USD per month (*n* = 181, 86.2%).

**Table 1:** Socio-demographic characteristics of the study participants (*n* = 420).

Variables	Dental students (*n* = 210)		Non-dental students (*n* = 210)	
	*n* (%)	Mean (SD)	*n* (%)	Mean (SD)
Gender				
Male	128 (61.0)		76 (36.2)	
Female	82 (39.0)		134 (63.8)	
Age		22.07 (2.66)		21.16 (1.20)
Economic status				
Below 350 USD	188 (89.5)		181 (86.2)	
350–599 USD	17 (8.1)		24 (11.4)	
600–1,000 USD	4 (1.9)		4 (1.9)	
Above 1,000 USD	1 (0.5)		1 (0.5)	

*n*, number; SD, standard deviation.

The association between the importance of an attractive face and the group of participants given in Table 2. Only the shape of the nose had a significant association with the group (*P* = 0.038). The dental students were more inclined to consider the shape of their nose as little important (13.3%) and important (58.6%) compared to the non-dental students (6.7%) and (55.7%), respectively. While the non-dental students were more inclined to consider the shape of their nose very important (36.7%) compared to the dental students (27.2%).

**Table 2:** The association between the importance of an attractive face and the group of participants.

Variables	Dental students *n* (%)	Non-dental students *n* (%)	Chi-square (DF)	*P*-value
Face			5.10 (3)	0.161
Not important	0 (0)	4 (1.9)		
Little important	10 (4.8)	7 (3.3)		
Important	70 (33.3)	61 (29.1)		
Very important	130 (61.9)	138 (65.7)		
Shape of face			1.70 (2)	0.427
Little important	18 (8.6)	13 (6.2)		
Important	115 (54.7)	109 (51.9)		
Very important	77 (36.7)	88 (41.9)		
Smile			0.07 (2)	1.000
Little important	8 (3.8)	7 (3.3)		
Important	97 (46.2)	98 (46.7)		
Very important	105 (50.0)	105 (50.0)		
Teeth			6.36 (3)	0.071
Not important	0 (0.0)	1 (0.5)		
Little important	5 (2.4)	0 (0.0)		
Important	72 (34.3)	79 (37.6)		
Very important	133 (63.3)	130 (61.9)		
Shape of nose			7.91 (3)	**0**.**038**
Not important	2 (0.9)	2 (0.9)		
Little important	28 (13.3)	14 (6.7)		
Important	123 (58.6)	117 (55.7)		
Very important	57 (27.2)	77 (36.7)		

F, frequency; DF, degree of freedom.

Bold indicates values that are statistically significant.

Based on the results indicated in Table 3, white teeth (*P* < 0.001), square oval-shaped teeth (*P* = 0.004), rectangular oval-shaped teeth (*P* = 0.007), and triangular oval-shaped teeth (*P* = 0.044) had a significant association with the study groups. The non-dental students were more inclined to strongly agree that they consider white teeth (71.1%), square oval-shaped teeth (64.9%), rectangular oval-shaped teeth (69.8%), and triangular oval-shaped teeth (68.1%) to be beautiful compared to the dental students. Whereas the dental students were more inclined to strongly agree that they consider aligned teeth (51.2%) as beautiful compared to the non-dental students; however, the association was not statistically significant (*P* = 0.933).

**Table 3:** The association between perceived beauty and the group of participants.

Parts	Dental students *n* (%)	Non-dental students *n* (%)	Chi-square (DF)	*P*-value
White teeth			27.17 (3)	**<0**.**001**
Strongly disagree	2 (100)	0 (0)		
Disagree	16 (80.0)	4 (20.0)		
Agree	166 (53.9)	142 (46.1)		
Strongly agree	26 (28.9)	64 (71.1)		
Aligned teeth			1.11 (3)	0.933
Strongly disagree	0 (0)	1 (100)		
Disagree	7 (46.7)	8 (53.3)		
Agree	137 (49.8)	138 (50.2)		
Strongly agree	66 (51.2)	63 (48.8)		
Square oval shape teeth			12.09 (3)	**0**.**004**
Strongly disagree	2 (66.7)	1 (33.3)		
Disagree	37 (67.3)	18 (32.7)		
Agree	151 (49.5)	154 (50.5)		
Strongly agree	20 (35.1)	37 (64.9)		
Rectangular oval shape teeth			11.73 (3)	**0**.**007**
Strongly disagree	3 (37.5)	5 (62.5)		
Disagree	78 (59.1)	54 (40.9)		
Agree	116 (48.9)	121 (51.1)		
Strongly agree	13 (30.2)	30 (69.8)		
Triangular oval shape teeth			7.46 (3)	**0**.**044**
Strongly disagree	1 (33.3)	2 (66.7)		
Disagree	37 (52.9)	33 (47.1)		
Agree	157 (52.3)	143 (47.7)		
Strongly agree	15 (31.9)	32 (68.1)		

F, frequency; DF, degree of freedom.

Bold indicates values that are statistically significant.

The results in Table 4 shows no significant association between the perceived importance of dental aesthetics and groups *(P* = 0.589). However, the dental students were more inclined to strongly agree that dental aesthetics are important (42.4%) compared to the non-dental students (39.5%).

**Table 4:** The association between the perceived importance of dental aesthetics and the group of participants.

Group	Dental aesthetics is important to me					
	Strongly disagree *n* (%)	Disagree *n* (%)	Agree *n* (%)	Strongly agree *n* (%)	Chi-square (DF)	*P*-value
Dental students	2 (1.0)	3 (1.4)	116 (55.2)	89 (42.4)	1.89 (3)	0.589
Non-dental students	1 (0.5)	1 (0.5)	125 (59.5)	83 (39.5)		

F, frequency; DF, degree of freedom.

The results in Table 5, indicate no significant association between the groups (*P* > 0.05). Nonetheless, the non-dental students show that there were more inclined to strongly agree that they consider relationships (58.6%), business or employment (62.0%), and friendship (56.5%) to be important for appearance compared to the dental students.

**Table 5:** The association between the perceived importance of appearance and the group of participants.

Variables	Dental students *n* (%)	Non-dental students *n* (%)	Chi-square (DF)	*P*-value
Relationship			4.95 (3)	0.138
Strongly disagree	1 (50.0)	1 (50.0)		
Disagree	13 (41.9)	18 (58.1)		
Agree	160 (53.3)	140 (46.7)		
Strongly agree	36 (41.4)	51 (58.6)		
Business/employment			6.34 (3)	0.074
Strongly disagree	2 (100)	0 (0)		
Disagree	23 (52.3)	21 (47.7)		
Agree	158 (52.1)	145 (47.9)		
Strongly agree	27 (38.0)	44 (62.0)		
Friendship			3.98 (3)	0.264
Strongly disagree	8 (66.7)	4 (33.3)		
Disagree	48 (56.5)	37 (43.5)		
Agree	127 (48.7)	134 (51.3)		
Strongly agree	27 (43.5)	35 (56.5)		
Self-confidence			0.94 (2)	1.000
Disagree	0 (0)	1 (100)		
Agree	97 (50.3)	96 (49.7)		
Strongly agree	113 (50.0)	113 (50.0)		
Facial appearance			2.77 (3)	0.459
Strongly disagree	0 (0)	3 (100)		
Disagree	2 (50.0)	2 (50.0)		
Agree	132 (50.2)	131 (49.8)		
Strongly agree	76 (50.7)	74 (49.3)		

F, frequency; DF, degree of freedom.

The Table 6 indicate that satisfaction with anterior tooth colour (*P* = 0.007), perception of poorly aligned teeth (*P* = 0.009), stains in front teeth (*P* < 0.001), and hiding teeth when smiling (*P* = 0.012) had a significant association with group. The dental students were more satisfied with anterior tooth color (55.9%) compared to the non-dental students (44.1%). In addition, the non-dental students were more inclined to report strongly agreeing with perceived poorly aligned anterior teeth (71.4%), stains in front teeth (81.3%), and always trying to hide teeth while smiling (58.6%) compared to the dental students.

**Table 6:** The association between perceived satisfaction with teeth and the group of participants.

Satisfaction	Dental students *n* (%)	Non-dental students *n* (%)	Chi-square (DF)	*P*-value
Are you satisfied with the appearance of your anterior teeth?			6.89 (3)	0.064
Very dissatisfied	4 (100)	0 (0)		
Dissatisfied	34 (42.0)	47 (58.0)		
Satisfied	142 (52.6)	128 (47.4)		
Very satisfied	30 (46.2)	35 (53.8)		
Are you satisfied with your anterior tooth color?			11.27 (3)	**0**.**007**
Very dissatisfied	1 (25.0)	3 (75.0)		
Dissatisfied	45 (41.3)	64 (58.7)		
Satisfied	151 (55.9)	119 (44.1)		
Very satisfied	13 (35.1)	24 (64.9)		
Do you feel your anterior teeth are poorly aligned e.g., crowded/spaced, rotated or not?			11.46 (3)	**0**.**009**
Strongly disagree	14 (73.7)	5 (26.3)		
Disagree	77 (53.5)	67 (46.5)		
Agree	109 (49.1)	113 (50.9)		
Strongly agree	10 (28.6)	25 (71.4)		
Do you have stains on your front teeth?			18.49 (3)	**<0**.**001**
Strongly disagree	29 (69.0)	13 (31.0)		
Disagree	109 (55.1)	89 (44.9)		
Agree	69 (42.1)	95 (57.9)		
Strongly agree	3 (18.8)	13 (81.3)		
Do you try to hide your teeth when you smile?			8.78 (2)	**0**.**012**
Never	91 (59.5)	62 (40.5)		
Sometimes	107 (45.0)	131 (55.0)		
Always	12 (41.4)	17 (58.6)		

F, frequency; DF, degree of freedom.

Bold indicates values that are statistically significant.

According to Table 7, previous history of whitening teeth (*P* < 0.001), orthodontic treatment (*P* = 0.003), aesthetic restorations (*P* = 0.009), and complete or partial removable dentures (*P* < 0.001) had a significant association with the group. The non-dental students reported a higher rate of previous whitening teeth (66.9%), aesthetic restorations (53.9%), and complete or partial removable dentures (72.4%) compared to the dental students. Whereas the dental students reported a higher rate of previous orthodontic treatment (58.4%) compared to the non-dental students.

**Table 7:** The association between previous treatment of anterior teeth and the group of participants.

Treatment	Dental students *n* (%)	Non-dental students *n* (%)	Chi-square (DF)	*P-*value
Whitening of teeth			20.87 (1)	**<0**.**001**
No	168 (57.3)	125 (42.7)		
Yes	42 (33.1)	85 (66.9)		
Orthodontic treatment			8.78 (1)	**0**.**003**
No	106 (43.8)	136 (56.2)		
Yes	104 (58.4)	74 (41.6)		
Dental crowns/bridge or veneers			0.06 (1)	0.812
No	164 (49.7)	166 (50.3)		
Yes	46 (51.1)	44 (48.9)		
Esthetic restorations			6.86 (1)	**0**.**009**
No	70 (60.3)	46 (39.7)		
Yes	140 (46.1)	164 (53.9)		
Complete or partial removable Denture			13.52 (1)	**<0**.**001**
No	194 (53.6)	168 (46.40		
Yes	16 (27.6)	42 (72.4)		
Implant			1.70 (1)	0.193
No	187 (51.2)	178 (48.8)		
Yes	23 (41.8)	32 (58.2)		

F, frequency; DF, degree of freedom.

Bold indicates values that are statistically significant.

The association between oral habits and the group of participants presented in Table 8, and the results indicate that frequency of brushing (*P* < 0.001), flossing teeth (*P* < 0.001), frequency of flossing teeth (*P* < 0.001), and frequency of dental visits (*P* < 0.001) had a significant association with group. The non-dental students were more inclined to brush their teeth three times a day (70.5%) compared to the dental students (29.5%). Whereas the dental students were more inclined to floss their teeth (59.0), floss their teeth several times a week (59.8%), and visit a dentist once every six months (73.3%) compared to the non-dental students.

**Table 8:** The association between oral habits and the group of participants.

Oral habits	Dental students *n* (%)	Non-dental students *n* (%)	Chi-square (DF)	*P*-value
Do you brush your teeth?			–	–
No	0 (0)	0 (0)		
Yes	210 (50.0)	210 (50.0)		
If yes, how often			33.36 (3)	**<0**.**001**
Less than once a day	0 (0)	3 (100)		
Once a day	3 (50.0)	3 (50.0)		
Twice a day	171 (59.2)	118 (40.8)		
Three times a day	36 (29.5)	86 (70.5)		
Do you floss your teeth?			39.09 (1)	**<0**.**001**
No	26 (24.1)	82 (75.9)		
Yes	184 (59.0)	128 (41.0)		
If yes, how often			19.34 (3)	**<0**.**001**
Less than once a week	22 (41.5)	31 (58.5)		
Several times a week	55 (59.8)	37 (40.2)		
Once a day	81 (73.0)	30 (27.0)		
More than once a day	26 (46.4)	30 (53.6)		
Do you ever go to the dentist?			–	–
No	0 (0)	0 (0)		
Yes	210 (50.0)	210 (50.0)		
If yes, how often			65.14 (4)	**<0**.**001**
Only when I have a problem	25 (26.0)	71 (74.0)		
Once every 6 months	110 (73.3)	40 (26.7)		
Once every year	27 (45.8)	32 (54.2)		
Once every few years	6 (20.7)	23 (79.3)		
Others	42 (48.8)	44 (51.2)		

F, frequency; DF, degree of freedom.

Bold indicates values that are statistically significant.

The association between treatment intentions and the group of participants are presented in Table 9. The results indicate that intention to undergo whitening of teeth (*P* < 0.001), aesthetic restorations (*P* = 0.019), dentures (*P* = 0.002), and stain removal (*P* = 0.011) had a significant association with the group. The non-dental students were more inclined to have the intention to undergo whitening of teeth (59.5%), aesthetic restorations (53.4%), dentures (68.3%), and stain removal (54.1%) compared to the dental students.

**Table 9:** The association between treatment intentions and the group of participants.

Do you wish to undergo any of these treatments to improve the appearance of your teeth?	Dental students *n* (%)	Non-dental students *n* (%)	Chi-square (DF)	*P*-value
Whitening of teeth			26.89 (1)	**<0**.**001**
No	101 (66.9)	50 (33.1)		
Yes	109 (40.5)	160 (59.5)		
Orthodontic treatment			1.94 (1)	0.163
No	55 (44.7)	68 (55.3)		
Yes	155 (52.2)	142 (47.8)		
Crowns/bridge or veneers			0.11 (1)	0.744
No	150 (49.5)	153 (50.5)		
Yes	60 (51.3)	57 (48.7)		
Esthetic restorations			5.46 (1)	**0**.**019**
No	65 (59.6)	44 (40.4)		
Yes	145 (46.6)	166 (53.4)		
Denture			9.88 (1)	**0**.**002**
No	190 (53.2)	167 (46.8)		
Yes	20 (31.7)	43 (68.3)		
Stain removal			6.42 (1)	**0**.**011**
No	77 (59.2)	53 (40.8)		
Yes	133 (45.9)	157 (54.1)		

F, frequency; DF, degree of freedom.

Bold indicates values that are statistically significant.

The Findings in Table 10 shows that non-dental students scored significantly higher (*M* = 11.78, SD = 1.87) than dental students (*M* = 11.17, SD = 1.61), *t* (418) = −3.56, *p* < 0.001. This makes it possible that the non-dental students have a greater awareness, knowledge, or use of the construct being measured. While dentists might have predicted the higher-level scoring for their group, the data fails to confirm inappropriate expectations regarding a professional education.

**Table 10:** The effect of socio-demographic factors on mean appearance satisfaction.

Variables	Mean ± SD/r	t/F (DF)	*P*
Group		−3.56 (418)	**<0** **.** **001**
Dental students	11.17 ± 1.61		
Non-dental students	11.78 ± 1.87		
Gender		1.51 (418)	0.131
Male	11.61 ± 1.81		
Female	11.35 ± 1.72		
Age (*r*)	0.093		0.056
Economic status		0.11 (3, 419)	0.956
Below 350 USD	11.46 ± 1.78		
350–599 USD	11.59 ± 1.69		
600–1,000 USD	11.50 ± 2.00		
Above 1,000 USD	11.00 ± 0.00		

SD, standard deviation; *r*, correlation coefficient; DF, degree of freedom.

Bold indicates values that are statistically significant.

Gender also did not result in any statistically significant differences, as male students (*M* = 11.61, SD = 1.81) and female students, (*M* = 11.35, SD = 1.72) performed identically, *t* (418) = 1.51, *p* = 0.131. Gender does not explain variation; similarly, there is little meaningful or uniform attitudes or knowledge between female and male participants.

Age and the outcome had a weak positive correlation (*r* = 0.093, *p* = 0.056), though it was approaching significance. Further, while the information implies older students scored somewhat higher than younger students, it makes sense that age is not a significant factor in performance.

In the end, there were no statistically significant differences in economic status groups, with mean average scores of 11.46 ± 1.78 (under 350 US dollars), 11.59 ± 1.69 (350–599 US dollars), 11.50 ± 2.00 (600–1,000 US dollars), and 11.00 ± 0.00 (above 1,000 US dollars); F(3,419) = 0.11, *p* = .956. This implies that a student's economic background did not differ significantly in terms of contributing towards results, and furthermore, that the students in each economic income group possessed comparably similar levels of performance.

## Discussion

Attitudes and perceptions towards dental appearance differ among populations and among individuals in a population (4). Patient satisfaction and aesthetic concern are essential factors that must be considered for successful dental treatment (5, 18). Our study investigated satisfaction with dental appearance, importance of dental appearance, participants' dental behaviors, previous dental treatment, and desire for treatment to improve dental appearance.

Perceptions of dental appearance differs between individuals and populations (16). Perceptions and attitudes regarding the appearance of the smile vary from one individual to another, and they are influenced by factors that affect the individuals in different ways, depending on their age, gender, marital status, socioeconomic status, level of education, occupation, the influence of family, peers, colleagues, cultural aspects and the mass media (16, 19). It has been suggested that many factors such as gender, socioeconomic background, age, education, cultural aspect, and family influence affect the self-perception of dental appearance (13). In our study, 64.3% of participants were satisfied with their anterior teeth' appearance. There were 67.6% of dental students who were satisfied with their anterior teeth appearance, compared with only 61% of non-dental students. Previous studies in different populations show different levels of satisfaction among their study participants. For example, 50% in Saudi Arabia (16), 47.2% in Malaysia (4), more than 56.3% in Nigeria (36), 64.9% in Jordan (37), 69.6% in Sudan (38), and 76% in United Kingdom (20). This could be attributed to using different measures to evaluate satisfaction, culture factors, religion, social, psychological, economic, individual characteristics, and racial factors that can affect dental appearance (4, 16).

### Dental appearance and color perception

Perception towards dental appearance is determined by cultural factors and individual preferences varying between individuals and cultures and changing over time (4). In our study, we found that 30.5% of non-dental students strongly agreed that white teeth are beautiful while only 12.4% of dental students thought that. In our study it was observed that dental students were more inclined to strongly agree that dental aesthetics are important (42.4%) compared to the non-dental students (39.5%). Analyzing the results, it was observed that non-dental students were more inclined to strongly agree that they consider relationships (58.6%), business or employment (62.0%), and friendship (56.5%) to be important for appearance compared to the dental students. Previous study that was done by Zavanelli and colleagues in the year 2017 found that 47.8% thought that dental appearance was important for relationship, 41.2% thought that dental appearance was important for business, and 11.0% thought that dental appearance was important in friendship (3).

Majority of participants 270 (64.3%) were satisfied with the anterior teeth color. It was seen that the non-dental students were more satisfied with anterior tooth colour (53.8%) compared to the dental students (46.2%) which was similar with the study that was done by Strajnić and team in the year 2016. Maghaireh and colleagues in the year 2016 found more than 50% of the participants from each age group were satisfied with their tooth color (17, 21), and also similar to other studies (4, 22), which was in contrasts with the findings of another study that reported a high level of dissatisfaction (15, 16).

There were 127 (30.2%) participants had done tooth whitening on their teeth, and we observed that 40.5% of the non-dental students' group had received tooth whitening before, while only 20% of dental students group had received that treatment before.

We observed that 76.2% of non-dental students wished to get tooth whitening more than the dental students' group (51.9%) did. Teeth whitening was observed to be desired treatment by participants to improve their anterior tooth color in the way of improving their dental appearance, which supports the idea of impact of tooth color on satisfaction with dental appearance, and it also coincides with a previous study (4, 16). A study that was done by Zavanelli and team in the year 2017 showed that 85.0% of the participants desired to undergo teeth whitening (4), 51.9% of the participants in the study that was done by Tin-Oo and team in the year 2011 desired to receive teeth whitening as well, 80.9% from 220 university students (16), and 55.3% of 450 patients who attended a dental teaching center in Jordan (17).

It is normal to note that, dental students understand the color characteristic of the dental structure, the physiological process of color change and their demand for tooth whitening (13). The higher rating of the ideal smile by dental students can be attributed to their theoretical and clinical background in relation to dental esthetics and their understanding of the dental factors affecting the smile (23).

Satisfaction with tooth color was found to be significantly related to the satisfaction with dental appearance. The finding supports the study by Tin-Oo and team in the year 2011, that satisfaction with tooth color impact on satisfaction with dental appearance (4). Moreover, stain removal was found to be desired treatment to improve their dental appearance by participants who felt that they had stains on their anterior teeth. A very significant finding was observed where 164 (39%) participants agreed that they had stains on their front teeth which could impact their appearance. On the other hand, 45.2% of non-dental students agreed that they have stains on their anterior teeth, while there were only 32.9% of the dental participants agreed with that. Previous study that was done by Meng and team in the year 2008, showed that people who recovered from the problems of stained teeth were reported improved satisfaction with their dental appearance (24). In addition, the non-dental students were more inclined to report strongly agreeing with perceived poorly aligned anterior teeth (71.4%) and always trying to hide teeth while smiling (58.6%) compared to the dental students. Previous study that was done by Tin-Oo and team found that only 32.3% of the participants feel their teeth were poorly aligned (4). Only 28.9% of the participants hiding their teeth during smiling in the study that was done by Maghaireh and colleagues (17). This could be attributed to the difference in sample size and used measures to evaluate satisfaction, psychological factors, age, educational, religious, and sociocultural factors. Several studies found that patients' satisfaction with their dental appearance was affected by tooth color (4, 5, 16, 18, 22).

### Treatment needs

We found that non-dental students (78.1%) had esthetic restoration; more than the dental students (66.7%). Previous study by Maghaireh and team in the year 2016 found that 39.8% of the participants received esthetic restoration before (17).

There was a significant number of participants, 311 (74%), who wanted to get esthetic restoration treatment to improve the appearance of their teeth; within all of that participant, there were 166 (79%) of non-dental students which was more than dental participant group, 145 (69%). The previous study that was carried out by Maghaireh and colleagues in the year 2016 showed that 37.3% of the participants desired for esthetic restoration (17). Another study that was done by Enabulele and Omo in the year 2017 found that 20.4% of the participants desired for tooth-colored filling (25). The study that was done by Zavanelli and team in the year 2017 found that 82.7% of the participants desired to undergo restoration (3). Another study that was done by Meng and colleagues in the year 2008 showed that participants who recovered from the problem such as a broken tooth or cap and those who did not have such a problem at each interview were more likely to report improved satisfaction with dental appearance (24).

There were a significant number of participants that (42.4%), had received orthodontic treatment before, and there were more dental students, 104 (49.5%) had received orthodontic than the non-dental students, 74 (35.2%). This supports previous research that states tooth alignment is essential in dento-gingival aesthetics (26). Malocclusion affects facial appeal, thus influencing self and social perception of adolescents (27). There is a strong correlation between dental treatment needs, especially esthetic treatments, and psychological satisfaction with dental appearance, that is affected by poor tooth color and alignment (4, 16). More than half of participants (52.9%) feel that their anterior teeth were poorly aligned e.g., crowded, spaced, or rotated. It contrasted with the result done in Nigeria by researchers Enabulele and Omo (84.7%) that most participants did not feel their teeth crowded (25).

### Esthetic needs and demands

We found that 64.3% of the participants were satisfied with their dental appearance, which shows that age relates to satisfaction with dental appearance since our participants were adults in university. However, it contrasts with the result of a previous study that found more satisfaction with dental appearance among older participants (20, 22). This variation may be attributed to the use of different measures to evaluate satisfaction, cultural factors, religion, social, psychological, economic, individual characteristics, and racial factors can affect dental appearance (4, 16). Self-perceived minor irregularities in dental esthetics might considerably impact OHRQoL (28). Different samples, measuring techniques, individual characteristics, psychology, and cultural, religious, and racial backgrounds might explain this controversy regarding the relationship between gender and satisfaction with dental appearance and tooth color (15).

The presence of poor tooth alignment, crowding, spacing, and the rotated tooth can be related to the level of satisfaction with dental appearance. Poor teeth alignment, crowded or spaced, rotated teeth, and stained anterior teeth could change the appearance of anterior teeth, causing them to be less attractive and can lead to patients trying to hide their teeth when they smile. In our study, we found that 56.7% of participants sometimes tried to hide their teeth when they smile. In a previous study by Al-Saleh and team in the year 2018, more than 40% of participants had the habit of covering their teeth while smiling (13). This result contrasts with other studies that showed that a high percentage of participants were comfortable with their smiles (14). These results could be explained by the effect of culture and society on individual self-perceptions (13). Hiding teeth while smiling is a reflection of dissatisfaction; further, the major goal of dental treatment should be to restore esthetics and enable patients to feel confident about smiling instead of hiding their teeth (18, 28).

### Oral health behaviors

Based on our findings, we noted that the majority of the dental students (81.4%) self-reported brushing their teeth two times per day (vs. 56.2% for the non-dental students), however 41% of non-dental participants brushed their teeth 3 times per day (vs. 17.1% of dental students). We reported that the most participants (87.6%) self-reported using floss (vs. 61% of non-dental students), but less participants (38.6%) responded to floss once a day (compared with 14.3% of non-dental students). In this study, we found that more than half (52.4%) of dental students had regular checkups once every six months, and only 19% of non-dental students had regular checkups once every six months. There were less than half (33.8%) of non-dental students went to meet the dentist only when they had a problem, while only 11.9% went to meet the dentist when they had a problem.

In the context of this study, the non-dental students' preoccupation with tooth whitening perhaps is associated with exposure to media promoting the beauty standard of bright white smiles as well as limited dental knowledge to other aspects of dental aesthetics (25). Cultural and social contexts such as peer reference and trends likely foster some concerns regarding whitening. While dental students’ training provides them with a more clinical understanding of smile aesthetics redefining their priorities. Although not measured here, there is sufficient likelihood to offer a plausible explanation that is consistent with current literature and thus needs further investigation (26).

## Strengths and limitations

The strengths of this study include the high response rate of participants who were students in the University of Puthisastra. The participants are probably representative of health science students in Cambodia.

However, this study was limited to testing satisfaction with dental appearance and desired dental treatment decisions. Therefore, the results of this study do not represent older age groups and cannot be generalized to the whole population. Questionnaire studies and surveys are always susceptible to bias as the data were collected in person. In situations where personal questions are asked e.g., in regards to brushing habits/ oral hygiene etc. there is always a risk of positivity bias as the patients may respond with exaggerated responses which can affect the findings of the study. Some of the data collected with regards to brushing habits, maintenance of oral hygiene and treatment undergone previously did not match up to expected results which could be considered surprising considering the existing situation (27).

Another limitation is the gender imbalance between the groups in which there were 61% male participants in the dental student group and 64% female participants in the non-dental student group. It is likely that the imbalance in gender played a role in their aesthetic preferences since there are gender-related perceptions of dental aesthetics.

Further, long-term longitudinal studies are required to evaluate the effect of age, level of education, income, social status, culture, and different conditions (physical and psychological) on satisfaction with dental appearance and the psycho-social impact of dental aesthetics. It would also be helpful to carry out similar studies across other regions or multi-cultural studies to analyze the effect of the local environment/education/cultural on the esthetic needs and demands of the participants there (18, 28).

Satisfaction with the appearance of teeth might be affected by cultural, social, psychological, economic, or religious factors in different populations might affect. Further studies are required to identify the potential effects of such factors in this regard.

## Conclusions

Based on the findings of our study we found that both dental and non-dental students show importance towards their esthetics. Dental education appears to have some influence on perceptions and attitudes between dental and non-dental students regarding their esthetic needs, current conditions and need for improved smiles. The majority of both dental and non-dental students are unhappy with their dental appearance, and wish to have a variety of treatments to improve this situation. Non-dental students wanted to get treatments to improve their dental problems and their dental appearance. This study demonstrated differences in opinion and knowledge regarding esthetics and dental treatment option demands between dental and non-dental students. Throughout the course of this study, we observed many differences in esthetic demands among dental and non-dentally educated students which could probably be attributed to the difference in the field specific education of the dental students.

The differing esthetic preferences between dental and non-dental students observed in this research imply that dentists must change the ways they communicate and plan treatment. In this case, non-dental patients may benefit from a greater understanding of realistic outcomes and clinical variables that contribute to smile esthetics, as it may help them align expectations with any achievable outcomes. A large part of a good treatment outcome is a patient's ability to understand the process they are involved in, and by integrating Visual aids and patient-centered discussions, dentists can improve patients' understanding and expectation, also providing opportunities for optimal collaboration with their dentist.

## Data Availability

The original contributions presented in the study are included in the article/Supplementary Material, further inquiries can be directed to the corresponding authors.
